# A Longitudinal Study of Parent-Child Interactions and Language Outcomes in Fragile X Syndrome and Other Neurodevelopmental Disorders

**DOI:** 10.3389/fpsyt.2021.718572

**Published:** 2021-11-08

**Authors:** Lauren Bush, Gary E. Martin, Emily Landau, Molly Losh

**Affiliations:** ^1^Roxelyn and Richard Pepper Department of Communication Sciences and Disorders, Northwestern University, Evanston, IL, United States; ^2^Department of Psychiatry and Behavioral Sciences, Autism Assessment, Research, Treatment, and Services Center, Rush University Medical Center, Chicago, IL, United States; ^3^Department of Communication Sciences and Disorders, St. John's University, Staten Island, NY, United States

**Keywords:** pragmatic language, social communication, fragile X syndrome, autism spectrum disorder, parent-child interaction, broad autism phenotype, longitudinal outcomes

## Abstract

Difficulties with pragmatic language (i.e., language in social contexts, such as conversational ability) are a noted characteristic of the language profiles of both fragile X syndrome (FXS) and autism spectrum disorder (ASD), conditions which show significant phenotypic overlap. Understanding the origins and developmental course of pragmatic language problems in FXS and other developmental conditions associated with language impairment is a critical step for the development of targeted interventions to promote communicative competence across the lifespan. This study examined pragmatic language in the context of parent-child interactions in school-age children with FXS (who did and did not meet ASD criteria on the ADOS; *n* = 85), idiopathic ASD (*n* = 32), Down syndrome (DS; *n* = 38), and typical development (TD; *n* = 39), and their parents. Parent-child communicative interactions were examined across multiple contexts, across groups, and in relationship to pragmatic language outcomes assessed 2 years later. Results showed both overlapping and divergent patterns across the FXS-ASD and idiopathic ASD child and parent groups, and also highlighted key differences in pragmatic profiles based on situational context, with more pragmatic language difficulties occurring for both ASD groups in less structured interactions. Differences in parental language styles during parent-child interactions were associated with child language outcomes, likely reflecting the complex interplay of discourse style inherent to a parent, with the inevitable influence of child characteristics on parent language as well. Together, findings help delineate the dynamic and multifactorial nature of impaired pragmatic skills among children with FXS and other neurodevelopmental disorders associated with language impairment, with potential implications for the development of targeted interventions for pragmatic communication skills.

## Introduction

Pragmatic language refers to the use of language in social contexts and draws on a broad range of linguistic, paralinguistic, neuropsychological, and social skills (1–10). For instance, successful conversations (a key pragmatic skill) require an individual to take turns; introduce, maintain, and change topics; demonstrate an awareness and understanding of conversational partners; and keep up with conversational demands and expectations (8). This dynamic and complex set of language skills also serves a pivotal role in supporting social interactions, and when impaired, can seriously undercut social functioning (6, 11).

Difficulties in pragmatic communication are a hallmark of autism spectrum disorder (ASD), a neurodevelopmental disability characterized by the presence of social and communicative impairments and patterns of restricted and repetitive behaviors or interests (12). Similar deficits are also observed in fragile X syndrome (FXS), a single-gene disorder caused by a mutation in the *FMR1* gene and the most common single-gene disorder associated with ASD (13–16). Importantly, areas of pragmatic language overlap (and divergence) have been noted between individuals with FXS-associated ASD (FXS-ASD) (based on meeting ASD criteria on the Autism Diagnostic Observation Schedule; ADOS) and idiopathic ASD (ASD-O) (17). For example, research has shown that both groups use non-contingent (i.e., off-topic) and perseverative (i.e., repetitive) language at higher rates than children with other types of neurodevelopmental disabilities, such as Down syndrome (DS) and FXS without significant ASD symptomatology (FXS-Only; FXS-O) (17–21). However, prior research has also found higher rates of initiations and lower rates of non-responsiveness in boys with FXS-ASD compared to boys with ASD-O (17). Evidence of similarities in pragmatic language phenotypes in FXS-ASD and ASD-O is potentially significant for understanding the shared etiology of such impairments, whereas knowledge of both similarities and differences can also inform pragmatic language interventions with these groups where targeted therapies can be implemented. Thus, clarifying the specific pragmatic needs of individuals with FXS with and without ASD symptomatology, and in relation to ASD-O, has important implications for targeting and advocating for more effective treatments in FXS.

Importantly, little is known about the development of pragmatic language in FXS compared to idiopathic ASD, and how conversational context and communication partner may contribute to patterns of pragmatic strengths and weaknesses—all critical questions to address in order to understand the extent of similarities in pragmatic profiles in FXS-ASD and ASD-O, and whether they may stem from common origins. Moreover, studies comparing pragmatic impairments in other genetically-based neurodevelopmental disorders (e.g., DS and FXS-O) relative to FXS-ASD and ASD-O are limited, leaving unclear the specificity of pragmatic impairments in these different populations (17, 22, 23). For example, pragmatic language impairments in DS may manifest differently due to a prominent discrepancy between social motivation and language difficulties (24–26). Evidence suggests that although children with DS commit fewer pragmatic violations compared to children with ASD and FXS (27–29), this group tends to show difficulty with topic elaboration, introduction, and maintenance (29, 30). They also tend to use increased stereotyped language compared to mental age matched controls (31, 32). Direct comparison of pragmatic language in DS and FXS with and without ASD symptomatology, and idiopathic ASD is needed to understand the specificity or potential overlap of such impairments across conditions, and to clarify the role of global developmental delay and intellectual disability (ID) vs. ASD-specific symptomatology in the pragmatic difficulties observed across groups. Indeed, while pragmatic language difficulties have been documented in ID, the nature of these impairments differs from what is seen in ASD, as social communication difficulties in ID are not thought to exceed the individual's broader profile of abilities and functional capacities (33).

This study adopted a cross-population, longitudinal design to comprehensively characterize pragmatic skills in school-age children with FXS who did and did not meet ASD criteria on the ADOS (FXS-ASD, FXS-O), idiopathic ASD (ASD-O), DS, and younger controls with typical development (TD), and to examine pragmatics across structured and unstructured conversational interactions with their parents. Importantly, parent-child communicative interactions served as the focus of analysis because parents are often the child's primary conversational partner, serving as a key source of language input throughout childhood. This is particularly true among children with developmental disabilities, as children with significant cognitive and language delays are less likely to extend the range of their communication partners throughout development relative to their typically developing counterparts (34, 35). Indeed, as children enter middle childhood and adolescence, they begin to master conversational skills, including appropriate referencing, increased turn-taking, adapting speaking style to conversational partner and context, and cohesion (36). Beyond this, the social demands of conversational contexts become more complex and nuanced over this developmental period (37), with an increased reliance on cognitive systems that are often significantly impacted in children with both ASD and ID. Studies of TD have also provided clear evidence of the critical role that parent discourse style can play in child language and social-emotional development [e.g., (38–41)]. In atypical development, parental discourse during parent-child interactions similarly has the potential to influence a child's language outcomes (42–45). Not surprisingly, overall parent-child synchrony and parental responsiveness during interactions is associated with better language outcomes in children with ASD (43, 46). Maternal responsiveness is also associated with child language in FXS (47). In addition, better pragmatic language in mothers of children with ASD appears related to better expressive language skills in 2–4-year-old children (45). Together, findings highlight the important ways in which parental language styles can influence children's language development, but also certainly reflect a bidirectional relationship in which parent and child language features influence each other in complex ways that have yet to be delineated in populations where pragmatics are centrally impacted. Most prior research addressing such questions has focused on early infancy and toddlerhood, leaving important questions unanswered regarding the school-age children, when continued and increasingly complex opportunities for language learning (particularly pragmatic language skills) and skill mastery occur (28, 48).

Examining parent-child interactions is also of particular significance in these groups given evidence of subtle pragmatic language differences among parents of individuals with ASD, which are believed to reflect genetic liability to ASD (20, 21, 49–53). Together with certain personality styles (e.g., social reticence, rigid personality), pragmatic differences comprise a constellation of traits that mirror the defining characteristics of ASD and are referred to collectively as the Broad Autism Phenotype (BAP) (20, 53). Features of the BAP (and pragmatic differences in particular) have also been observed among mothers of individuals with FXS, who are carriers of the *FMR1* gene in its premutation state (20, 54). Some evidence suggests that parent pragmatic language differences are associated with pragmatic language development in children with ASD and FXS (45, 55, 56). To date, however, no study has directly examined the potential association between parent and child pragmatic language during parent-child interactions in school-age children, and the majority of studies that have looked at parent-child relationships have relied on global measures of language from separate conversational contexts in parents and their children, rather than the parent-child interactions in which such associations might be most effectively studied. Data examining the interrelationships between parents and children during conversational interactions has the potential to identify key pragmatic features in both communicative partners that can serve as important pathways for targeting parent-mediated interventions in order to most effectively address the child's particular needs. Similarly, delineating the complexity of these relationships may also highlight BAP features that serve as protective factors on child language development (e.g., eliciting more language by indulging tangents; adhering to routine-based interactions). Thus, this approach can also clarify parents' pragmatic strengths in order to maximize these in treatment and optimize child outcomes.

This study applied a detailed hand-coding (i.e., manual, turn-by-turn coding) system adapted from Roberts et al. (29) and Martin et al. (17), and previously used to describe pragmatic language in children with FXS, ASD, DS, and TD during semi-structured interactions with a trained examiner, to characterize pragmatic language across groups during two distinct parent-child interaction contexts, with a subgroup studied over time. In addition to group comparisons, analyses examined interrelationships between parent and child pragmatic phenotypes, and how such features related to child pragmatic outcomes 2 years later. The overarching goals of this study were to delineate the complex pragmatic language phenotypes associated with different neurodevelopmental conditions and identify the influence of parent-child interaction styles on child language outcomes across these groups. Specific aims were as follows:

**Aim 1: To compare child pragmatic profiles across groups during parent-child interactions**. Key pragmatic language features were compared across groups. Sex differences were also examined. Based on the extant literature, alongside underlying difficulties with social cognition observed in ASD, it was predicted that the ASD groups (FXS-ASD and ASD-O) would demonstrate greater pragmatic deficits relative to the comparison groups, with most profound differences noted in key areas of non-contingent and perseverative language (20–22, 28, 29, 57). It was further predicted that individuals in the ASD groups would demonstrate better pragmatic abilities during structured interaction as compared to unstructured interaction given the greater social demands inherent in unstructured situations, and evidence suggesting that unstructured discourse contexts are most challenging for individuals with ASD [e.g., (58)].
**Aim 2: To compare parent pragmatic profiles across groups during parent-child interactions**. Given evidence of pragmatic language differences in the broad autism phenotype and among a subgroup of carriers of the *FMR1* premutation, and alongside weaknesses in social cognition, it was predicted that parents of children in the ASD groups (FXS-ASD and ASD-O) would exhibit greater differences in pragmatic behaviors, including non-contingent language, which has been reported in prior literature. The effects of context were predicted to mirror the same trends that were expected for children.
**Aim 3: To examine interrelationships between parent and child language**. It was expected that non-contingent and perseverative language would be interrelated in parents and children in all groups, but specifically the ASD groups.
**Aim 4: To identify key features of parent-child interactions that predict child pragmatic outcomes 2 years later, across diagnostic groups**. Overall parent and child responsiveness during parent-child interactions was predicted to influence child pragmatic language outcomes across groups (44, 47, 59).

## Materials and Methods

Parent and child participants were recruited as part of a larger study on pragmatic language development. Both males and females were included in all groups except the idiopathic ASD group, which included only males due to the aims of the larger longitudinal study from which these data were drawn (see Table 1 for participant characteristics). Although the majority of the parent sample consisted of mothers, fathers participated in 17 cases across groups (3 fathers participated with males with FXS-ASD, 5 with males with ASD-O, 3 with males with FXS-O, 2 with males with DS, 2 with males with TD, and 1 with a female with TD). Mothers in the FXS groups were all confirmed carriers of the *FMR1* premutation. The total parent sample therefore consisted of 17 father-child dyads and 177 mother-child dyads. Sixteen sets of siblings were included in which a parent participated more than one time with a different child in the same diagnostic group. To address this, the effect of family was examined in statistical analyses and is reported in the analysis plan below. All siblings and parents were included in the overall sample.

**Table 1:** Participant characteristics.

**Males**					
**Variable**	**FXS-ASD,** **M (SD)** ***n* = 39**	**FXS-O,** **M (SD)** ***n* = 10**	**ASD-O,** **M (SD)** ***n* = 32**	**DS,** **M (SD)** ***n* = 21**	**TD,** **M (SD)** ***n* = 19**
Chronological age	10.9 (2.3)^a^	9.6 (3.2)^a, b^	8.6 (2.8)^b^	**10.9 (2.1)** ^**a**^	4.6 (1.1)^c^
Non-verbal mental age^a^	5.1 (0.41)^a^	**5.3 (0.63)** ^**a**^	7.0 (3.5)^b^	5.1 (0.41)^a^	5.2 (1.2)^a^
Receptive vocabulary age^b^	5.9 (1.4)^a, b^	**5.9 (1.6)** ^**a, b**^	6.3 (2.7)^a^	5.9 (1.4)^b^	6.0 (1.6)^a^
Expressive vocabulary age^c^	**5.2 (1.0)** ^**a, c**^	**5.2 (1.0)** ^**a, b**^	6.0 (2.3)^b^	5.4 (1.3)^c^	5.6 (1.6)^d^
MLU^d^	**3.4 (0.76)** ^**a**^	**3.9 (0.75)** ^**a**^	4.1 (1.1)^b^	3.1 (0.77)^a^	4.8 (0.73)^b^
Autism severity^e^	6.6 (1.5)^a^	2.5 (1.0)^b^	7.6 (1.9)^c^	1.4 (0.55)^b^	1.6 (0.69)^b^
Parent education level	16.0 (2.4)^a^	15.9 (1.3)^a^	16.0 (2.3)^a^	16.6 (2.3)^a^	16.0 (2.5)^a^
**Females**					
**Variable**	**FXS-ASD**, **M (SD)** ***n*** **=** **11**	**FXS-O**, **M (SD)** ***n*** **=** **25**	**ASD-O**, **M (SD)** ***n*** **=** **32**	**DS**, **M (SD)** ***n*** **=** **17**	**TD**, **M (SD)** ***n*** **=** **20**
Chronological age	9.1 (3.8)^a^	9.0 (3.7)^a^		**9.1 (2.2)** ^**a**^	5.4 (2.4)^b^
Non-verbal mental age^a^	5.4 (0.95)^a^	**7.0 (2.7)** ^**b**^		5.1 (0.71)^a^	6.2 (2.6)^a, b^
Receptive vocabulary age^b^	7.4 (3.4)^a^	**8.2 (3.5)** ^**a**^		4.7 (1.6)^b, c^	6.5 (3.1)^a, c^
Expressive vocabulary age^c^	**6.4 (2.0)** ^**a, b**^	**8.4 (3.8)** ^**b**^		4.7 (1.5)^a^	6.2 (2.4)^a^
MLU^d^	**4.3 (1.2)** ^**a**^	**4.8 (1.1)** ^**a**^		3.3 (1.0)^b^	5.0 (1.4)^a^
Autism severity^e^	6.1 (1.7)^a^	2.1 (0.86)^b^		1.8 (0.62)^b, c^	1.5 (0.67)^c^
Parent education level	15.0 (1.6)^a^	16.0 (2.1)^a^		15.4 (2.3)^a^	16.0 (2.8)^a^

*FXS-ASD, fragile X syndrome with autism spectrum disorder based on the ADOS; ASD-O, ASD only; FXS-O, FXS only; DS, Down syndrome; TD, typical development; M, Mean; SD, Standard Deviation. Different superscripts within a row indicate significant differences at p < 0.05. If groups share the same letter, differences were not significant. Bolded items indicate significant sex differences.*

*^a^Leiter International Performance Scale-Revised.*

*^b^Peabody Picture Vocabulary Test-III or IV.*

*^c^Expressive Vocabulary Test.*

*^d^Mean length of utterance in morphemes.*

*^e^Autism Diagnostic Observation Schedule*.

Inclusion criteria for the broader longitudinal study [described in greater detail in Ref. (17)] included English as a primary language, using three or more words in an utterance, having no history of developmental or language delays in the TD group, and having the *FMR1* full mutation in the FXS group. Participants who failed a hearing screening with a threshold >30 dB HL in the better ear across 500, 1,000, 2,000, and 4,000 Hz were excluded from the study. Participants in the TD and DS groups were screened for ASD using the Autism Diagnostic Observation Schedule (ADOS) (60). Any subject in the TD or DS groups who met criteria for autism or autism spectrum on the ADOS were excluded from the broader longitudinal study, while subjects with idiopathic ASD or FXS who met ADOS criteria for autism or autism spectrum were included in one of the ASD groups. Of note, the Autism Diagnostic Interview, Revised (ADI-R) (61) was administered whenever possible, though due to time constraints was available on only 56% of the sample. Therefore, the ADOS was used for group classification. Participants in the ASD-O group had all previously received a clinical diagnosis of ASD. Because subjects received multiple administrations of the ADOS as part of the larger longitudinal research study, average ADOS severity scores were calculated to determine ASD classification [(62); see further description in Ref. (17)]. All examiners who administered the ADOS satisfied reliability criteria set forth by the test authors. Eight participants were excluded from the present study (and are thus not included in Table 1) because the dyad either interacted for <5 min and/or did not speak long enough to generate the minimum number of total turns required for analyses (20 turns for each 5-min task). For the structured task, this included 1 male with FXS-ASD, 2 females with FXS-ASD, and 1 female with DS. For the unstructured task, this included 2 males with ASD-O, 1 female with FXS-ASD, and 1 female with FXS-O.

Participants enrolled in this study were administered a battery of language, cognitive, and clinical-behavioral measures in addition to the ADOS. The larger study from which these data were drawn implemented a rolling enrollment schedule, where participants were eligible to enroll at any point during the 5-year study period. Participants who enrolled later in the study did not complete later timepoints of longitudinal data collection, but were nonetheless included at time 1 to increase power for group comparison data. The total sample size for longitudinal analyses included: 28 boys and 8 girls with FXS-ASD, 5 boys and 21 girls FXS-O, 11 boys with ASD-O, 14 boys and 10 girls with DS, and 9 boys and 9 girls with TD.

The study battery was administered in a quiet room, either in the child's home, school, or at a research laboratory. Testing sessions were audiotaped using a digital audio recorder (Marantz PMD670) and videotaped using a SONY Digital 8 camcorder (Model DCR-TVR27). All procedures were approved by University of North Carolina at Chapel Hill and Northwestern University Institutional Review Boards.

### Cognitive and Structural Language Abilities

The Leiter International Performance Scale-Revised (63) was used to assess non-verbal cognitive abilities. Structural language measures included expressive vocabulary, receptive vocabulary, and expressive syntactic complexity. These skills were assessed using the Expressive Vocabulary Test [EVT; (64)], Peabody Picture Vocabulary Test-3rd or 4th edition [PPVT; (65, 66)], and mean length of utterance (MLU) in morphemes (67), respectively. MLU was based on ADOS language samples, which occurred at the same time point as the parent-child interactions and were computed using Systematic Analysis of Language Transcripts [SALT; (68)] software.

### Parent-Child Interactions

Parent-child interactions included one structured and one relatively unstructured interactive task, each lasting 5-min.

#### Structured Interaction

Parents and children were asked to plan a “fun day out” together. For this task, the examiner provided five different picture cards and instructed parent-child dyads to discuss where they would like to go, who they would like to go with, what they would need to bring, how they would get there, and what they would like to do/see at the destination. The picture cards involved scenes from the zoo, park/playground, pool, beach, and shopping center.

#### Unstructured Interaction

Parents and children engaged in a “free play” task in which examiners presented a box of toys (e.g., flashlight, kaleidoscope, prism, rainbow glasses, periscope, picture cards) and provided the parent and child with only minimal instructions to look at the toys together, so that interactions could unfold in a relatively unstructured manner.

### Pragmatic Language

Pragmatic language was measured both at study entry, and 2 years following the initial parent-child interaction, using the following measures.

#### Comprehensive Assessment of Spoken Language (CASL)

The Pragmatic Judgment subtest of the CASL (69) is a standardized test measure that requires children to state how they would respond in various social situations (e.g., “How would you greet an unfamiliar adult?”). Consistent with past research [e.g., (70)], age equivalence scores were used in analysis.

#### Pragmatic Rating Scale-School Age (PRS-SA)

The Pragmatic Rating Scale-School Age [PRS-SA; (71)] is a pragmatic language rating system designed to characterize a range of pragmatic language abilities based on semi-naturalistic, conversational interactions administered as part of the ADOS. The PRS-SA includes 34 operationally defined verbal and non-verbal pragmatic language features rated 0, 1, or 2 (indicating presence and degree of impairment for each item, with scores of 2 indicating greatest impairment) by independent coders from videotaped recordings. Coders were never provided with participant diagnostic status, but were also unlikely to be fully blinded given facial dysmorphology that occurs in DS and often FXS. Reliability for the PRS-SA based on the larger study from which these data were drawn, as well as ongoing studies in our lab which include samples not included in the current study, is 78.4%. A subset of these files were consensus coded, and the consensus coded scores were used in analyses.

### Transcription and Coding

Parent-child language samples were transcribed verbatim by transcribers who achieved morpheme-to-morpheme agreement rates of 80% or higher. Coders (coding system described below) were similarly trained to a minimum of 80% training reliability across three separate files. As was the case with the PRS-SA, fully blinded status of coders was not possible given the use of video. Nine percent of all coded files were also randomly checked for reliability. Intraclass correlation coefficients [ICCs; (72)] were as follows for children: non-contingent language (0.94), perseveration (0.87), initiations (0.98), and non-responsiveness (0.87). ICCs were as follows for parents: non-contingent language (0.84), perseveration (0.68), initiations (0.99), and non-responsiveness (1.0). ICCs from 0.5 to 0.75 are considered to represent moderate agreement, 0.75–0.9 to represent good agreement, and >0.9 to represent excellent agreement (73). The reliability files were subsequently consensus coded. Coding was based on parent-child dyadic turns. A parent-child dyadic turn was defined as either one back-and-forth parent-child exchange (e.g., parent speaks and child responds or vice versa) and/or a comment/question that was met with a non-response by the other conversational partner for a period of at least 3 s. Each parent-child task was coded separately, by coders who were blind to group status.

### Pragmatic Language Coding System

Pragmatic language skills during the structured and unstructured interaction tasks were coded using a system adapted from Roberts et al. (29) and Martin et al. (17), which examines discrete aspects of pragmatic language, such as contingency of conversational partners' contributions, initiations and responsiveness, and perseverative language. Pragmatic codes are further described in Table 2. Unintelligible utterances were excluded from calculations.

**Table 2:** Pragmatic coding system.

**Variable**	**Definition**	Example (s)*	**Calculation**
Non-contingent Language	Off-topic or tangential turns	Par: What is this? **Ch: Good to see you**.	Total non-contingent turns/total codable dyadic turns
Perseveration	Excessive repetition of words, phrases, sentences, or topics	Par: What's in there? Ch: I ain't telling. Par: I wanna see. Ch: She always lets me look. Nobody else. Par: I'm gonna cry. I'm gonna cry. Ch: She's supposed to let me look. Par: I'm gonna cry. Do you want me to cry? Ch: She always let's me look in here. Par: Okay, but do you want me to cry? Ch: No. Par: Well I am going to cry if you don't let me look. … Ch: She told me I can look in here. She is a student and she told me I can look. Par: I am going to cry now. C: That's what she said. P: I think I'm going to cry now.	Total perseverative turns/total codable dyadic turns
Initiations	Self-initiated turns	Par: I think we would need to take the car to the zoo. Ch: Yea, it would be a long walk if we didn't drive. **What animals will be there?**	Total initiations/total codable dyadic turns
Non-response	Failure to respond when response is required (within 3 s)	Par: What do we do with this toy? **Ch: No response**.	Total non-responses/total codable dyadic turns

*With the exception of perseveration, which demonstrates examples for both parent and child, examples demonstrate each variable in the child, with the same coding guidelines used for parents.

### Analysis Plan

Analyses of group differences in children and parents controlled for child non-verbal mental age, expressive and receptive vocabulary, and mean length of utterance (MLU), given significant differences across groups and because of the impact of general cognition and structural language on pragmatic abilities [e.g., (74)]. There were no significant differences in parental education across groups where data were available (*n* = 55; ps > 0.80) and thus this was not included as a covariate. Given that participants were classified based only the ADOS, partial correlations for participants with FXS (regardless of ASD classification status) were conducted using the four child outcome variables and ASD severity. Results based on these analyses are presented as a complementary table (see Table 3.4) and allow for examination of relationships with ASD severity as a continuous measure. Across analyses (with the exception of linear regressions for interrelationships and correlational analyses described in pragmatic longitudinal outcomes), planned comparisons were conducted even when overall models were not significant, given the novelty of this data and to guard against Type 2 errors (75). In addition, given multiple tests and a rather small dataset, Bonferroni corrected analyses were conducted for child and parent MANCOVAs following initial analyses with no adjustments, to address the possibility of false discovery. Only findings that withstood Bonferroni corrections are reported in the text below. These findings are also denoted in each of the corresponding tables. However, because adjustment assumes that a Type 1 error is of more serious concern than a Type 2 error (76), and given the uniqueness of these data and difficulty ascertaining rare populations such as FXS (particularly for longitudinal studies), the danger of missing effects was of greater concern. Therefore, findings without adjustments for the MANCOVAs are also reported in tables. To further aid in interpretation of data, Cohen's d effect sizes are also provided for all analyses examining group differences (see Tables 3.3, 4.3). With the exception of pragmatic longitudinal outcomes which include both significant and marginal findings given small sample size, only significant results are described. The corresponding tables for each analysis present the remaining statistical results.

#### Pragmatic Language in Children

Pragmatic language during parent-child interactions was analyzed using a series of multivariate analyses of covariance (MANCOVAs). Group differences were examined, as well as within group sex differences (except for the ASD-O group, where data on females were not available). The effect of context (structured vs. unstructured parent-child interaction task) was evaluated using repeated measures ANCOVA to investigate a diagnosis by context interaction.

#### Pragmatic Language in Parents

To characterize parent pragmatic profiles across groups during parent-child interactions, analyses followed the same plan that was used for the analysis of child data described above (i.e., group comparisons were based on child diagnosis and sex). To address concerns related to non-independence (i.e., a small subset of cases included the same parent with a different child, *n* = 16), a linear mixed model was conducted for each outcome variable with participant nested within family. None of these models were significant and the random effect for diagnosis in these cases was essentially zero. This suggested that the nesting of individuals within families did not result in non-independence. While it is possible that there was an insufficient number of family cases included in the overall sample to fully test this effect, generalized linear models (GLMs) were additionally conducted with and without siblings and the findings were compared to each other. Because there were few differences between these two models, and there was no effect of family status in the mixed model, the GLMs reported below include all available participants regardless of family status in order to increase sample size and power.

#### Parent-Child Interrelationships

To examine patterns of parent-child relationships, and limit the number of correlations examined, a principal component analysis (PCA) with a one component solution was conducted for parent and child groups separately with all language variables (i.e., non-contingent language, non-responsiveness, initiation, perseveration) included. The PCA resulted in a component for the child group explaining 40.09% of the variance, with standardized loadings of 0.26 for initiation, 0.37 for non-responsiveness, 0.84 for non-contingent language, and 0.84 for perseveration. The parent component explained 35.37% of the variance, with standardized loadings of −0.39 for initiation, 0.32 for non-responsiveness, 0.82 for non-contingent language, and 0.70 for perseveration. The component score for each subject was then used in exploratory Pearson correlations within each group, with separate analyses for sex and context, resulting in a total of 18 correlation models.

#### Pragmatic Language Outcomes

To identify the relationship between parent-child interactions (including both child and parent language during interactions) at baseline and child pragmatic outcomes 2-years later across diagnostic groups, a series of partial correlations were conducted. The variables included each of the parent and child language variables explored in the group differences and interrelationship analyses above (e.g., parent and child non-contingent language), as well as PRS-SA and CASL scores at time 3 (two years later). Covariates included the baseline measure of the outcome variable being explored, which was mean centered (e.g., PRS-SA scores at baseline served as the covariate for relationships with longitudinal PRS-SA scores). Of note, all parents of boys with ASD-O and girls with FXS-ASD included in longitudinal analyses received scores of zero for non-responsiveness, resulting in insufficient variability within these groups to examine these relationships in a meaningful way and relationships with parental non-responsiveness for these two groups were therefore not examined.

## Results

### Pragmatic Language in Children

#### Boys

As indicated in Table 3.1, the model for non-contingent language was significant in both tasks, driven by boys with FXS-ASD and ASD-O compared to all other groups. There were no significant effects of context. Similar findings emerged for perseveration, such that boys with FXS-ASD and ASD-O used higher rates of perseveration compared to other groups during structured interactions. Non-responsiveness was significant during both interaction types. This effect was driven by higher rates of non-responsiveness among boys with ASD-O compared to all other groups. Boys with FXS-ASD, ASD-O, DS, and TD were more non-responsive during the unstructured interactions compared to the structured interactions.

**Table 3.1:** Group differences in pragmatic language in males.

**Structured interaction**						
**Pragmatic language**	**Group F**	**FXS-ASD,** **M(SE)**	**FXS-O,** **M(SE)**	**ASD-O,** **M(SE)**	**DS,** **M(SE)**	**TD,** **M(SE)**
Non-contingent	*F*_(4, 112)_ = 10.7^***^^†^	0.18 (0.02)^a^	0.08 (0.03)^b^	0.16 (0.02)^a^	0.04 (0.02)^b^	0.07 (0.02)^b^
Perseveration	*F*_(4, 112)_ = 6.1^***^^†^	0.07 (0.009)^a^	0.03 (0.02)^b^	0.05 (0.01)^a, b^	0.005 (0.01)^c^	0.02 (0.01)^c^
Initiations	*F*_(4, 112)_ = 1.3	0.34 (0.02)^a, b^	0.42 (0.04)^a^	0.31 (0.03)^b^	0.33 (0.03)^a, b^	0.34 (0.03)^a, b^
Non-responsiveness	*F*_(4, 112)_ = 7.3^***^^†^	***0.01 (0.006)** ^***a***^*	0.01 (0.01)^a^	**0.05 (0.007)** ^**b**^	**0.003 (0.008)** ^**a**^	**0.02 (0.009)** ^**a**^
**Unstructured interaction**						
**Pragmatic language**	**Group F**	**FXS-ASD**, **M(SE)**	**FXS-O**, **M(SE)**	**ASD-O**, **M(SE)**	**DS**, **M(SE)**	**TD**, **M(SE)**
Non-contingent	*F*_(4, 112)_ = 16.2^***^^†^	*0.24 (0.02)^*a*^*	0.07 (0.03)^b^	0.18 (0.02)^a^	0.05 (0.03)^b^	0.06 (0.03)^b^
Perseveration	*F*_(4, 112)_ = 4.2**	0.11 (0.01)^a^	0.04 (0.03)^b^	0.07 (0.02)^a, b^	0.03 (0.02)^b^	0.03 (0.02)^b^
Initiations	*F*_(4, 112)_ = 0.19	0.49 (0.02)^a^	0.53 (0.04)^a^	0.49 (0.03)^a^	0.48 (0.03)^a^	0.49 (0.04)^a^
Non-responsiveness	*F*_(4, 112)_ = 6.7^***^^†^	***0.04 (0.02)** ^***a***^*	0.03 (0.03)^a^	**0.14 (0.02)** ^**b**^	**0.04 (0.02)** ^**a**^	**0.08 (0.02)** ^**a**^
**Context effect**						
**Pragmatic language**	**Group F**					
Non-contingent	*F*_(4, 112)_ = 1.4					
Perseveration	*F*_(4, 112)_ = 0.38					
Initiations	*F*_(4, 112)_ = 0.45					
Non-responsiveness	*F*_(4, 112)_ = 2.6*					

*FXS-ASD, fragile X syndrome with autism spectrum disorder based on the ADOS; ASD-O, ASD only; FXS-O, FXS only; DS, Down syndrome; TD, typical development. Adjusted Means (M) and Standard Errors (SE). Different superscripts within a row indicate significant differences at p < 0.05. If groups share the same letter, differences were not significant. Bolded items indicate significant differences between contexts within a diagnostic group. Italicized items indicated significant within group sex differences.*

*p < 0.05, **p < 0.01, ***p < 0.001,^†^indicates significance following Bonferroni corrections.

#### Girls

Girls with FXS-ASD used more non-contingent language relative to all other groups (see Table 3.2). There were no significant effects for context. Similar findings emerged for perseveration and were primarily driven by girls with FXS-ASD and FXS-O. There was no significant effect for context overall. Girls with FXS-ASD were less responsive during structured and unstructured interactions than girls in all other groups. There was no significant effect for context.

**Table 3.2:** Group differences in pragmatic language in females.

**Structured interaction**					
**Pragmatic language**	**Group F**	**FXS-ASD,** **M(SE)**	**FXS-O,** **M(SE)**	**DS,** **M(SE)**	**TD,** **M(SE)**
Non-ontingent	*F*_(3, 65)_ = 6.2^**^^†^	0.17 (0.02)^a^	0.06 (0.02)^b^	0.04 (0.02)^b^	0.05 (0.02)^b^
Perseveration	*F*_(3, 65)_ = 5.4^**^^†^	0.05 (0.01)^a^	0.03 (0.007)^a, c^	−0.003 (0.009)^b^	0.007 (0.008)^b, c^
Initiations	*F*_(3, 65)_ = 0.85	0.38 (0.05)^a^	0.41 (0.03)^a^	0.37 (0.04)^a^	0.41 (0.04)^a^
Non-responsiveness	*F*_(3, 65)_ = 5.4^***^^†^	*0.06 (0.009)^*a*^*	0.01 (0.006)^b^	0.002 (007)^b^	0.02 (0.007)^b^
**Unstructured interaction**					
**Pragmatic language**	**Group F**	**FXS-ASD**, **M(SE)**	**FXS-O**, **M(SE)**	**DS**, **M(SE)**	**TD**, **M(SE)**
Non-contingent	*F*_(3, 65)_ = 8.4^***^^†^	0.15 (0.02)^a^	0.06 (0.02)^b^	0.03 (0.02)^b^	0.04 (0.02)^b^
Perseveration	*F*_(3, 65)_ = 6.1^**^^†^	0.10 (0.02)^a^	0.07 (0.01)^a, b^	−0.002 (0.02)^c^	0.04 (0.02)^b, c^
Initiations	*F*_(3, 65)_ = 1.9	0.61 (0.05)^a^	0.61 (0.03)^a^	0.52 (0.04)^b^	0.52 (0.04)^a, b^
Non-responsiveness	*F*_(3, 65)_ = 3.3^*^^†^	*0.13 (0.02)^*a*^*	0.05 (0.02)^b^	0.03 (0.02)^b^	0.05 (0.02)^b^
**Context effect**					
**Pragmatic language**	**Group F**				
Non-contingent	*F*_(3, 65)_ = 0.49				
Perseveration	*F*_(3, 65)_ = 1.5				
Initiations	*F*_(3, 65)_ = 1.6				
Non-responsiveness	*F*_(3, 65)_ = 0.58				

*FXS-ASD, fragile X syndrome with autism spectrum disorder based on the ADOS; FXS-O, FXS only; DS, Down syndrome; TD, typical development. Adjusted Means (M) and Standard Errors (SE).Different superscripts within a row indicate significant differences at p < 0.05. If groups share the same letter, differences were not significant. Italicized items indicated significant within group sex differences.*

*p < 0.05, **p < 0.01, ***p < 0.001,^†^indicates significance following Bonferroni corrections.

**Table 3.3:** Effect sizes (Cohen's d) for pragmatic language in males and females.

**Structured interaction/unstructured interaction**						**Structured interaction/unstructured interaction**			
**Non-contingent language**	**Males**					**Females**			
	**FXS-ASD**	**FXS-O**	**ASD-O**	**DS**	**TD**	**FXS-ASD**	**FXS-O**	**DS**	**TD**
FXS-ASD	–	0.9/1.4	0.17/0.5	1.2/1.5	1.0/1.4	–	1.2/1.7	1.8/1.5	1.5/1.4
FXS-O	–	–	0.7/1.0	0.4/0.16	0.1/0.08	–	–	0.2/0.5	0.1/0.3
ASD-O	–	–	–	1.2/1.1	0.88/1	–	–	–	–
DS	–	–	–	–	0.3/0.07	–	–	–	0.1/0.1
**Perseveration**	**Males**					**Females**			
	**FXS-ASD**	**FXS-O**	**ASD-O**	**DS**	**TD**	**FXS-ASD**	**FXS-O**	**DS**	**TD**
FXS-ASD	–	0.7/1.0	0.4/0.5	1.2/1.1	0.97/1.1	–	0.6/0.6	1.5/1.4	1.3/0.8
FXS-O	–	–	0.4/0.3	0.5/0.1	0.2/0.1	–	–	0.9/1.1	0.7/0.4
ASD-O	–	–	–	0.9/0.4	0.6/0.4	–	–	–	–
DS	–	–	–	–	0.3/0.0	–	–	–	0.3/0.5
**Initiations**	**Males**					**Females**			
	**FXS-ASD**	**FXS-O**	**ASD-O**	**DS**	**TD**	**FXS-ASD**	**FXS-O**	**DS**	**TD**
FXS-ASD	–	0.7/0.3	0.2/0.0	0.08/0.8	0.0/0.0	–	0.2/0.0	0.06/0.6	0.2/0.5
FXS-O	–	–	0.7/0.3	0.7/0.4	0.6/0.3	–	–	0.3/0.6	0.0/0.6
ASD-O	–	–	–	0.1/0.06	0.2/0	–	–	–	–
DS	–	–	–	–	0.08/0.07	–	–	–	0.2/0.0
**Non-responsiveness**	**Males**					**Females**			
	**FXS-ASD**	**FXS-O**	**ASD-O**	**DS**	**TD**	**FXS-ASD**	**FXS-O**	**DS**	**TD**
FXS-ASD	–	0.0/0.09	1.1/0.85	0.2/0	0.3/0.4	–	1.7/0.9	2.1/1.4	1.3/1.1
FXS-O	–	–	1.1/1.0	0.2/0.1	0.3/0.6	–	–	0.3/0.3	0.3/0.1
ASD-O	–	–	–	1.2/0.9	0.8/0.5	–	–	–	–
DS	–	–	–	–	0.5/0.5	–	–	–	0.6/0.2

*Effect sizes were calculated based on adjusted mean, standard error, and individual group sample size. Cells with – indicate redundant effect sizes, within-group effect sizes that are not calculated, or effect sizes that could not be calculated (e.g., no ASD-O group females)*.

**Table 3.4:** Partial correlations based on ASD-severity and pragmatic language in FXS.

	**Non-contingent**		**Perseveration**		**Nonresponse**		**Initiations**	
	**Structured**	**Unstructured**	**Structured**	**Unstructured**	**Structured**	**Unstructured**	**Structure**	**Unstructured**
Males	0.44**	0.46**	0.30*	0.39**	0.002	0.01	−0.02	0.02
Females	0.53**	0.36*	0.16	0.05	0.5**	0.36*	−0.17	−0.00

Results based on correlational analyses generally follow the same trends as those observed based on group classification status; Bolded items indicate statistical significance; Italicized items indicated significant within group sex differences. *p < 0.05, **p < 0.01.

#### Sex Differences

There were significant sex differences in rates of non-responsiveness between boys and girls with FXS-ASD [*F*_(1, 46)_ = 13.8, *p* < 0.01; *F*_(1, 46)_ = 3.8, *p* < 0.01] across both interaction contexts, with girls showing higher rates of non-responsiveness than boys.

### Pragmatic Language in Parents

#### Parents of Boys

Parents interacting with their children with FXS-ASD and ASD-O used more non-contingent language across both contexts compared to parents from other groups (see Table 4.1). Parents of children with ASD-O and FXS-ASD were also more perseverative than parents interacting with children with DS. Rates of initiations and non-responsiveness were not significant for either context.

**Table 4.1:** Group differences in pragmatic language in parents of males.

**Structured interaction**						
**Pragmatic language**	**Group F**	**FXS-ASD,** **M(SE)**	**FXS-O,** **M(SE)**	**ASD-O,** **M(SE)**	**DS,** **M(SE)**	**TD,** **M(SE)**
Non-contingent	*F*_(4, 112)_ = 3.2^*^^†^	**0.02 (0.004)** ^**a**^	0.02 (0.008)^a, b, c^	**0.02 (0.005)** ^**a, c**^	0.002 (0.006)^b^	**0.006 (0.006)** ^**b, c**^
Perseveration	*F*_(4, 112)_ = 0.34	**0.01 (0.005)** ^**a**^	**0.02 (0.009)** ^**a**^	0.006 (0.005)^a^	0.007 (0.007)^a^	0.009 (0.007)^a^
Initiations	*F*_(4, 112)_ = 0.11	0.95 (0.008)^a^	0.94 (0.02)^a^	0.95 (0.009)^a^	0.95 (0.01)^a^	0.95 (0.01)^a^
Non-responsiveness	*F*_(4, 112)_ = 0.34	0.006 (0.007)^a^	−0.002 (0.01)^a^	0.002 (0.008)^a^	0.01 (0.009)^a^	0.003 (0.01)^a^
**Unstructured interaction**						
**Pragmatic language**	**Group F**	**FXS-ASD**, **M(SE)**	**FXS-O**, **M(SE)**	**ASD-O**, **M(SE)**	**DS**, **M(SE)**	**TD**, **M(SE)**
Non-contingent	*F*_(4, 112)_ = 4.9^**^^†^	**0.06 (0.007)** ^**a**^	0.01 (0.01)^b^	**0.04 (0.008)** ^**a, b**^	0.02 (0.01)^b^	**0.01 (0.01)** ^**b**^
Perseveration	*F*_(4, 112)_ = 2.9^*^^†^	**0.05 (0.01)** ^**a**^	**0.03 (0.02)** ^**a, b**^	0.05 (0.01)^a^	0.004 (0.01)^b^	0.01 (0.02)^a, b^
Initiations	*F*_(4, 112)_ = 0.97	0.92 (0.01)^a^	0.92 (0.02)^a^	0.92 (0.01)^a^	0.91 (0.02)^a^	0.88 (0.02)^a^
Non-responsiveness	*F*_(4, 112)_ = 1.4	0.01 (0.01)^a^	0.003 (0.02)^a^	0.01 (0.01)^a^	0.04 (02)^a^	0.04 (0.02)^a^
**Context effect**						
**Pragmatic language**	**Group F**					
Non-contingent	*F*_(4, 112)_ = 2.2^∧^					
Perseveration	*F*_(4, 112)_ = 3.0*					
Initiations	*F*_(4, 112)_ = 1.4					
Non-responsiveness	*F*_(4, 112)_ = 0.79					

*Parent groups based on child status; FXS-ASD, fragile X syndrome with autism spectrum disorder based on the ADOS; ASD-O, ASD only; FXS-O, FXS only; DS, Down syndrome; TD, typical development. Adjusted Means (M) and Standard Errors (SE). Different superscripts within a row indicate significant differences at p < 0.05. If groups share the same letter, differences were not significant. Bolded items indicate significant differences between contexts within a diagnostic group.*

^∧^p < 0.10, *p < 0.05, **p < 0.01,^†^indicates significance following Bonferroni correction.

#### Parents of Girls

The FXS-ASD parent group used higher rates of non-contingent language relative to the other groups (see Table 4.2). Higher rates of non-contingent language were observed during unstructured interaction relative to the structured interaction in parents of individuals with FXS-O and DS. Non-responsiveness did not occur often enough during structured interactions for a valid model estimate to be derived.

**Table 4.2:** Group differences in pragmatic language in parents of females.

**Structured interaction**					
**Pragmatic language**	**Group F**	**FXS-ASD,** **M(SE)**	**FXS-O,** **M(SE)**	**DS,** **M(SE)**	**TD,** **M(SE)**
Non-contingent	*F*_(3, 65)_ = 2.6^∧^	0.02 (0.006)^a^	0.004 (0.004)^b^	0.004 (0.005)^b^	0.01 (0.005)^a, b^
Perseveration	*F*_(3, 65)_ = 0.76	0.005 (0.003)^a^	0.001 (0.002)^a^	0.00 (0.002)^a^	0.003 (0.002)^a^
Initiations	*F*_(3, 65)_ = 0.77	0.94 (0.02)^a^	0.95 (0.01)^a^	0.96 (0.01)^a^	0.94 (0.01)^a^
Non-responsiveness	–	0.00 (0.00)	0.00 (0.00)	0.00 (00)	0.00 (0.00)
**Unstructured interaction**					
**Pragmatic language**	**Group F**	**FXS-ASD**, **M(SE)**	**FXS-O**, **M(SE)**	**DS**, **M(SE)**	**TD**, **M(SE)**
Non-contingent	*F*_(3, 65)_ = 1.2	0.04 (0.01)^a^	0.02 (0.007)^a^	0.02 (0.01)^a^	0.02 (0.008)^a^
Perseveration	*F*_(3, 65)_ = 1.8	0.03 (0.01)^a^	0.008 (0.006)^b^	0.01 (0.008)^a, b^	0.009 (0.007)^a, b^
Initiations	*F*_(3, 65)_ = 0.29	0.93 (0.02)^a^	0.90 (0.02)^a^	0.91 (0.02)^a^	0.91 (0.02)^a^
Non-responsiveness	*F*_(3, 65)_ = 0.13	0.02 (0.02)^a^	0.02 (0.01)^a^	0.009 (01)^a^	0.01 (0.01)^a^
**Context effect**					
**Pragmatic language**	**Group F**				
Non-contingent	*F*_(3, 65)_ = 0.58				
Perseveration	*F*_(3, 65)_ = 1.4				
Initiations	*F*_(3, 65)_ = 0.77				
Non-responsiveness	*F*_(3, 65)_ = 0.13				

*Parent groups based on child status; FXS-ASD, fragile X syndrome with autism spectrum disorder based on the ADOS; FXS-O, FXS only; DS, Down syndrome; TD, typical development. Adjusted Means (M) and Standard Errors (SE). Different superscripts within a row indicate significant differences at p < 0.05. If groups share the same letter, differences were not significant. ^∧^p < 0.10*.

**Table 4.3:** Effect sizes (Cohen's d) for pragmatic language in parents.

**Structured interaction/unstructured interaction**						**Structured interaction/unstructured interaction**			
**Non-contingent language**	**Parents of males**					**Parents of females**			
	**FXS-ASD**	**FXS-O**	**ASD-O**	**DS**	**TD**	**FXS-ASD**	**FXS-O**	**DS**	**TD**
FXS-ASD	–	0/1.2	0/0.5	0.7/0.9	0.6/1.2	–	0.8/0.6	0.8/0.5	0.5/0.6
FXS-O	–	–	0/0.7	0.7/0.2	0.6/0	–	–	0/0	0.3/0
ASD-O	–	–	–	0.7/0.4	0.5/0.7	–	–	–	–
DS	–	–	–	–	0.2/0.2	–	–	–	0.3/0
**Perseveration**	**Parents of males**					**Parents of females**			
	**FXS-ASD**	**FXS-O**	**ASD-O**	**DS**	**TD**	**FXS-ASD**	**FXS-O**	**DS**	**TD**
FXS-ASD	–	0.5/0.3	0.1/0.4	0.1/0.7	0.1/0.5	–	0.4/0.7	0.5/0.6	0.1/0.7
FXS-O	–	–	0.6/0.3	0.6/0.5	0.5/0.3	–	–	0.1/0.1	0.3/0
ASD-O	–	–	–	0.03/0.7	0.1/0.6	–	–	–	–
DS	–	–	–	–	0.04/0.2	–	–	–	0.3/0
**Initiations**	**Parents of males**					**Parents of females**			
	**FXS-ASD**	**FXS-O**	**ASD-O**	**DS**	**TD**	**FXS-ASD**	**FXS-O**	**DS**	**TD**
FXS-ASD	–	0.2/0	0/0	0/0.1	0/0.6	–	0.2/0.3	0.3/0.6	0/0.3
FXS-O	–	–	0/0	0/0.1	0/0.5	–	–	0.2/0.1	0.2/0.1
ASD-O	–	–	–	0/0.1	0/0.6	–	–	–	–
DS	–	–	–	–	0/0.3	–	–	–	0.5/0
**Non-responsiveness**	**Parents of males**					**Parents of females**			
	**FXS-ASD**	**FXS-O**	**ASD-O**	**DS**	**TD**	**FXS-ASD**	**FXS-O**	**DS**	**TD**
FXS-ASD	–	0.2/0.1	0.1/0	0.1/0.4	0.1/0.4	–	–/0	–/0.2	–/0.2
FXS-O	–	–	0.1/0.1	0.3/0.5	0.1/0.5	–	–	–/0.2	–/0.2
ASD-O	–	–	–	0.2/0.4	0/0.4	–	–	–	–
DS	–	–	–	–	0.2/0	–	–	–	–/0

*Effect sizes were calculated based on adjusted mean, standard error, and individual group sample size. Cells with – indicate redundant effect sizes, within-group effect sizes that are not calculated, or effect sizes that could not be calculated (e.g., no ASD-O group in parents of females)*.

### Parent-Child Interrelationships

Correlation coefficients from the PCA-derived components are summarized in Table 5 and reported in detail here. In boys and girls with FXS-ASD, the components were associated between the parent and child groups in the unstructured interactions (rs > 0.61, *ps* < 0.01), and there were no significant associations in the structured interactions (*ps* > 0.08). No significant associations emerged in the FXS-O groups (*ps* > 0.28) or DS groups (*ps* > 0.16). For boys with ASD-O, a significant relationship emerged between the parent and child groups during structured interactions (*r* = 0.46, *p* = 0.01) but not in unstructured interactions (*r* = 0.05, *p* = 0.79). In girls with TD, a significant association emerged in unstructured interactions (*r* = 48, *p* = 0.03). There were no associations in boys with TD (*ps* > 0.20).

**Table 5:** Interrelationships between parent and child pragmatic language.

		**Child component**	
		**Structured context**	**Unstructured context**
**Parent Component**	FXS-ASD boys (*n* = 39)	0.27	0.62***
	FXS-ASD girls (*n* = 11)	0.47	0.75**
	FXS-O boys (*n* = 10)	0.22	0.30
	FXS-O girls (*n* =25)	0.07	−0.07
	ASD-O boys (*n* = 32)	0.46**	0.05
	DS boys (*n* = 21)	0.15	0.09
	DS girls (*n* = 17)	0.18	0.17
	TD boys (*n* = 19)	−0.01	0.27
	TD girls (*n* = 20)	0.18	0.48*

Bolded items indicate statistical significance; *p < 0.05, **p < 0.01, ***p < 0.001.

### Pragmatic Language Outcomes

#### FXS-ASD

**Figure 1:**
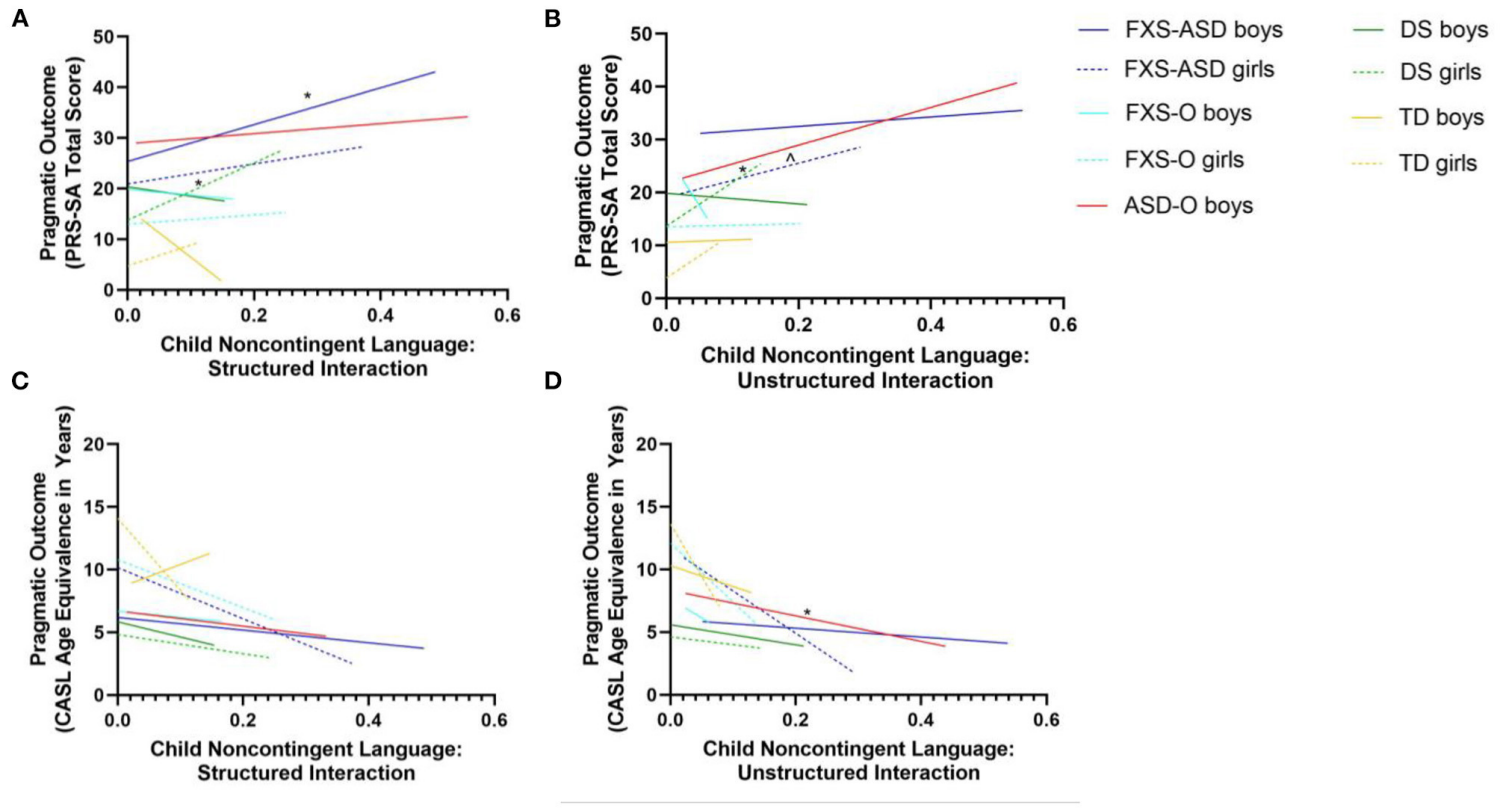
**(A–D)** Non-contingent language and pragmatic language outcomes. Associations between non-contingent language and pragmatic language outcomes. **(A)** Increased rates of non-contingent language in the structured interactions were significantly associated with poorer pragmatic language outcomes, as measured by the PRS-SA, in FXS-ASD boys and DS girls. **(B)** Increased use of non-contingent language in the unstructured interactions was correlated with poorer pragmatic language outcomes, as measured by the PRS-SA, in the FXS-ASD girls and DS girls. **(C)** There were no significant associations between non-contingent language and pragmatic language outcomes, as measured by the CASL, in any groups. **(D)** Increased rates of non-contingent language in the unstructured interactions was significantly associated with poorer pragmatic language outcomes on the CASL in boys with ASD. FXS-ASD, fragile X syndrome with autism spectrum disorder; FXS-O, FXS only; ASD-O, ASD only; DS, Down syndrome; TD, typical development. ^∧^*p* < 0.1, *p < 0.05.

Increased rates of child non-contingent language (*r* = 0.51, *p* = 0.007) and perseveration (*r* = 0.42, *p* = 0.03) in the structured interaction were associated with greater pragmatic difficulties in boys with FXS-ASD 2 years later, as measured by the PRS-SA (see Figure 1A). Increased rates of parent non-responsiveness (*r* = 0.34, *p* = 0.08) and parent perseveration (*r* = −0.35, *p* = 0.07) in the structured interaction were marginally associated with greater pragmatic difficulties in boys based on the PRS-SA. In contrast, among girls, increased rates of child non-contingent language in the unstructured interaction were marginally associated with better pragmatic outcomes based on the PRS-SA (*r* = −0.68, *p* = 0.09; see Figure 1B). No other significant or marginal relationships emerged (*p*s > 0.10) (e.g., see Figure 1C).

#### FXS-O

Increased rates of child perseveration (*r* = −0.96, *p* = 0.04) and child non-responsiveness (*r* = 0.98, *p* = 0.02) during structured parent-child interactions were associated with greater pragmatic difficulties 2 years later, as measured by the CASL, in boys with FXS-O. There were no significant relationships when examining the parent-child interactions and child longitudinal outcomes based on the PRS-SA in this group (*p*s > 0.17). In girls with FXS-O, higher rates of parent non-contingent language in the structured interaction (*r* = −0.48, *p* = 0.03) were associated with greater pragmatic difficulties, as measured by the CASL, 2 years later. Marginal relationships emerged between parent initiations in the unstructured interaction and pragmatic outcomes based on the PRS-SA (*r* = 0.41, *p* = 0.07). No other significant or marginal relationships emerged for girls with FXS-O on either the CASL or PRS-SA (*p*s > 0.14) (e.g., see Figure 1C).

#### ASD-O

Partial correlations in boys with ASD-O revealed that child non-contingent language in the unstructured interaction (*r* = −0.90, *p* = 0.001) was associated with lower (i.e., worse) child CASL scores 2 years later (see Figure 1D). Parent initiations in the unstructured interaction were negatively related to child pragmatic outcomes based on the CASL (*r* = −0.68, *p* = 0.05). Parent non-responsiveness in unstructured interaction was also related to better pragmatic outcomes on the CASL (*r* = 0.78, *p* = 0.01). No other significant relationships emerged (*p*s > 0.24) (e.g., see Figure 1C).

#### DS

In boys with DS, a marginal relationship emerged between child non-responsiveness in the unstructured interaction and pragmatic outcomes based on the PRS-SA (*r* = 0.49, *p* = 0.09). Child perseveration in the structured interaction (*r* = −0.53, *p* = 0.08) and parent non-responsiveness in the unstructured interaction (*r* = −0.54, *p* = 0.07) were also marginally related to CASL scores 2 years later, such that higher rates of child perseveration and parent non-responsiveness were associated with greater pragmatic difficulties 2 years later. In girls with DS, increased parent initiations in the structured interaction (*r* = −0.71, *p* = 0.02) and perseveration in the unstructured interaction (*r* = −0.63, *p* = 0.05) were related to poorer pragmatic outcomes, as measured by the CASL. However, increased child non-contingent language (rs > 0.74, *p*s <0.03) during both contexts and increased child perseveration in the unstructured context (*r* = 0.78, *p* = 0.01) was associated with higher (i.e., worse) PRS-SA scores (see Figures 1A,B). Marginal relationships also emerged between child initiations in the structured interaction and pragmatic outcomes on the PRS-SA (*r* = −0.61, *p* = 0.08) (i.e., increased initiations during parent-child interactions associated with better PRS-SA scores 2 years later). No other significant or marginal relationships emerged (*p*s > 0.11) (e.g., see Figure 1C).

#### TD

Partial correlations revealed a significant relationship between parent perseveration in the unstructured context (*r* = 0.89, *p* = 0.003) and CASL scores 2 years later in boys with TD, such that higher rates of parent perseveration were associated with better pragmatic outcomes. Higher rates of parent non-contingent language in the structured context (*r* = −0.72, *p* = 0.05) and non-responsiveness in the unstructured context (*r* = −0.71, *p* = 0.05) were also associated with better pragmatic scores on the PRS-SA in boys with TD. Marginal relationships emerged between child perseveration in the unstructured context in boys and pragmatic outcomes based on the CASL (*r* = 0.66, *p* = 0.08). Higher rates of child non-responsiveness in the unstructured context were associated with better pragmatic scores on the CASL in girls with TD (*r* = 0.83, *p* = 0.02), and increased parent perseveration in the unstructured contexts was marginally associated with more pragmatic difficulties on the PRS-SA (*r* = 0.67, *p* = 0.07). No other significant or marginal relationships emerged (*p*s > 0.10).

## Discussion

This study applied a detailed pragmatic coding system to characterize parent and child pragmatic skills during conversational interactions in children with fragile X syndrome who did and did not meet ASD criteria on the ADOS (FXS-ASD, FXS-O), idiopathic autism spectrum disorder (ASD-O), Down syndrome (DS), and typical development (TD), as well as interrelationships and pragmatic language outcomes 2 years later. Sex differences were also examined within each diagnostic group, with the exception of the ASD-O group because girls were not included in the broader longitudinal study from which these data were drawn. Results suggest both important areas of overlap and divergence in pragmatic skills and patterns of association, across child and parent groups, and in different conversational contexts. The FXS-ASD and ASD-O groups showed particular similarities, with some important exceptions. Parent-child analyses also suggest associations indicative of reciprocal interactions in the ways that parents and children use pragmatic skills during both structured and unstructured conversations, although patterns differed across groups, and direction of influence in these associations remains unclear (though likely to be highly bidirectional). In what follows, results are discussed in greater detail across each of the primary sets of analyses.

### Child Group and Sex Differences

In line with prior research where the child interacted with a trained examiner (17, 29), and consistent with predictions, boys and girls with FXS-ASD, and boys with ASD-O, demonstrated higher rates of non-contingent language during structured (i.e., “fun day out” task) and unstructured (i.e., “free play” task) parent-child interactions relative to all other groups. The findings for perseverative language generally paralleled the same trends that emerged for non-contingent responses (although the model for perseveration during unstructured interactions did not withstand multiple-comparison correction and should be interpreted with caution). In other words, boys and girls with FXS-ASD were more perseverative during both structured and unstructured interactions relative to all other groups. Boys with ASD-O were significantly more perseverative during structured interaction than boys with TD and DS. Although this finding was not significant during unstructured interaction, the same pattern of differences emerged. Together, these findings add to a growing body of research that supports non-contingent and perseverative language as central components of the pragmatic phenotype associated with ASD with and without FXS (17–19, 29, 77).

In contrast to these areas of overlap, we observed differences between the two groups of boys with ASD, particularly with respect to lower rates of initiations and responsiveness in boys with ASD-O compared to FXS-ASD (although the model for initiations did not withstand multiple-comparison correction and should be interpretated with caution). Nonetheless, this finding builds on those reported from a prior study looking at examiner-child interactions in an overlapping sample of participants (17), suggesting a more pervasive pattern that extends across different types of conversational interactions and partners. These findings may reflect important differences in underlying social motivations, as the ability to initiate conversation represents a core pragmatic impairment among boys with ASD-O (17, 78–81). Similarly, the ability to be responsive also represents a clear pragmatic deficit for boys with ASD-O and girls with FXS-ASD. This finding was expected and adds to the growing body of evidence pointing to non-responsiveness as a major factor in the pragmatic phenotype of idiopathic ASD (17, 82, 83). It also reveals an important sex difference between boys and girls with FXS-ASD, as boys did not show difficulty with responsiveness. Notably, social anxiety and hyperarousal are major factors in the social phenotype of girls with FXS-ASD (84), and could be contributing to these findings.

Important differences were also observed in boys and girls with FXS-O and DS. Consistent with recent research, neither of these groups had difficulty with non-contingent language relative to typically developing controls (17, 19, 29, 70), which suggests that increased non-contingent language use may be more unique to individuals in the ASD groups and is not alone attributable to general cognitive delay. In fact, contingent discourse represented a relative strength among boys and girls with FXS-O and DS. Additionally, inasmuch as previous investigations have suggested that non-contingent responding is typical of children with FXS [e.g., (85, 86)], it is important to note that defining the presence vs. absence of ASD symptomatology appears to make an important difference.

Whereas, boys and girls with DS did not show any difficulty with perseveration, girls with FXS-O were more perseverative than girls with DS or TD, and there were no differences in rates of perseveration between boys with FXS-O and ASD-O, who both showed more perseveration than DS and TD groups. These findings are slightly different from what was observed in a similar sample during examiner-child interactions, where Martin et al. (17) found no evidence of increased perseveration in the FXS-O groups. This important context difference highlights perseveration as a key behavior to consider in FXS, independent of ASD symptomatology.

### Parent Group and Sex Differences

The pragmatic profiles of parents generally followed the same trends that occurred for children. Parents interacting with girls and boys with FXS-ASD, and boys with ASD-O, showed higher rates of non-contingent language in both contexts relative to the other groups. Additionally, these same parent groups used higher rates of perseverative language during unstructured parent-child interactions (although the model for perseveration in parents of girls did not withstand multiple-comparison correction and should be interpretated with caution). It is possible that these findings were child-driven, as these parent groups have children who show the very same types of pragmatic weaknesses. However, these types of pragmatic differences have also been described as important features of the BAP (20, 21, 50, 53). A subgroup of parents of children with ASD and carriers of the *FMR1* premutation have been shown to use more tangential (including topic preoccupation) or off-topic language, during conversational interactions with examiners (20).

These findings suggest that non-contingent and perseverative language represent an important component of the pragmatic phenotype associated with ASD with and without FXS, in both affected and unaffected individuals. This potential pragmatic *signature* appears to cut across diagnostic boundaries in children and parents, and may help define the etiologies of pragmatic impairment in FXS and ASD. These findings also underscore the need to consider parental language style in the development of targeted parent-child interventions, as parents may be genetically predisposed to certain language styles that are exacerbated in the context of interactions with their children.

### Interrelationships Between Parent and Child Pragmatic Behaviors

A number of interesting patterns of parent-child interrelationships emerged across groups for the PCA-derived components. Non-contingent language and perseveration contributed most significantly to both the parent and child components (>0.70), with initiation and non-responsiveness contributing less so with standardized loadings <0.40. Higher ratings on both components indicated more difficulty with pragmatic language, specifically more non-contingent language and perseveration, during the dyadic interactions.

Examining these components, significant positive parent-child correlations emerged for boys and girls with FXS-ASD, boys with ASD-O, and girls with TD. For boys and girls with FXS-ASD and boys with ASD-O, increased rates of non-contingent language and perseveration were observed in analyses of group differences, with similar patterns emerging in their parents, likely reflecting similar pragmatic weaknesses in these dyads. The positive association further indicates that, within groups, parents with more severe weaknesses were more likely to have children with more severe weaknesses. This finding is potentially indicative of genetic influences, although environmental effects cannot be ruled out. The significant association for girls with TD and their parents is perhaps less notable given that pragmatic weaknesses were not found for these groups, although the correlation still indicates that such behaviors, even if relatively infrequent, are related in these dyads.

The bidirectional nature of these associations is important to consider, as parent and child language patterns are certainly interdependent. For example, a parent could be off-topic because their child was off-topic to begin with or vice versa, or non-contingent language could occur as part of a parents' natural attempt to redirect their child to a particular topic. It may also be that because of a child's tendency to respond in an off-topic or socially inappropriate way during conversational interactions, parents may be more prone to change the topic or redirect the conversation back to what was originally being discussed. In sum, parent and child behaviors are highly interrelated and in important ways driven by the other's behaviors. The bidirectional nature of these relationships should be further studied in designs capable of teasing out causal direction, such as parent training intervention studies, which may help to identify the most effective response patterns for supporting the development of more contingent discourse in these dyadic interactions.

Together, these findings emphasize the ways in which parent and child contributions to an unfolding communicative interaction are intricately interrelated. It is clear that even if parents and children have genetic predispositions toward certain language styles, pragmatic features do not occur in isolation; instead, they are dynamic and bidirectional. Children who have difficulty with these types of pragmatic skills in particular could benefit from targeted interventions aimed at improving specific parent-child interaction patterns.

Finally, the lack of significant parent-child interrelationships in boys or girls with FXS-O and DS, and boys with TD, may be due to fewer atypical pragmatic behaviors being present in these groups overall. This may also suggest that dyads in these groups employed different pragmatic styles, perhaps inconsistently, that did not significantly relate to one another.

### Pragmatic Language Outcomes

Results from longitudinal analyses suggest meaningful parent and child language variables as potential factors related to long-term child pragmatic outcomes, with slightly different patterns emerging across groups. For example, higher rates of non-contingent and perseverative language were associated with greater pragmatic difficulties in boys with FXS-ASD. Not surprisingly, and similar to boys with FXS-ASD, a relationship between increased non-contingent language and poorer pragmatic language outcomes also emerged among boys with ASD-O. This suggests that child non-contingent language during parent-child interactions in both ASD groups predicts greater pragmatic difficulty later in development. These findings highlight an important target for intervention efforts in both ASD groups, and may suggest the ways in which this shared aspect of the pragmatic phenotype in idiopathic and syndromic ASD contributes to developmental outcomes. While the opposite relationship emerged for girls with FXS-ASD, it should be noted that this was of marginal significance. Interestingly, in boys with FXS-O, increased rates of child perseverative and non-responsive language were associated with greater pragmatic deficits 2 years later. Consistent with the literature, this finding suggests that regardless of ASD status, perseveration likely represents an important target in pragmatic language interventions for children with FXS (87), as results indicate it may be related to a broader pattern of pragmatic language difficulties over time. In the context of these findings, clarifying the presence of ASD symptomatology in individuals with FXS has important implications for developing tailored treatment plans aimed at improving pragmatic language outcomes, with areas of meaningful clinical overlap and divergence in the development of treatment goals.

In girls with FXS-O, higher rates of parental non-contingent language during parent-child interactions were associated with greater pragmatic difficulties 2 years later. While not statistically significant, marginal relationships emerged in a similar direction between parental non-responsiveness and perseveration in boys with FXS-ASD. While larger samples and increased power may have yielded a clearer pattern of results, these findings provide an initial indication that parent language style during parent-child interactions may contribute to the child's pragmatic outcomes, even during the school-age years, highlighting an important clinical target that could be addressed in future parent-child intervention studies in FXS.

In contrast, a slightly different pattern emerged in boys with ASD-O, such that increased rates of parent initiations were associated with poorer pragmatic language outcomes on the CASL, a standardized measure of pragmatic language 2 years later. A similar finding was seen among girls with DS. Notably, the content and quality of the parents' initiations in these cases are not entirely clear, although it may be the case that overly frequent parental initiations occurred at the expense of reciprocal parent-child communication. Alternatively, particularly among the ASD-O group, it is also quite possible that parents over-initiated as a way to compensate for their child's non-responsive behaviors, which may thus serve as a mediating factor in this complicated, transactional relationship.

### Study Strengths, Limitations, and Clinical and Research Directions

An important contribution of this study was the inclusion of multiple clinical groups included with individuals with FXS. This afforded analysis of pragmatic language profiles that may be unique to FXS, as well as how pragmatic abilities in children with FXS might be influenced by ASD symptoms. Examining language samples in two different contexts, in relationship to parental pragmatic language, and over time offered additional, rich information to further specify pragmatic language abilities and developmental outcomes in FXS. Finally, this study is among the first in to examine the impact of parent-child interactions in school-age children in FXS, ASD, and DS, as the majority of work in this area has focused on toddlers and preschool-age children.

An important limitation of the study is the reliance on the ADOS as the single method of ASD classification. Most individuals with FXS had not been clinically evaluated for ASD previously, and due to time restrictions and participant retention considerations, the ADI-R could only be administered to roughly half the sample, necessitating reliance on the ADOS for group classification [see also (88)]. Future studies should rely on multiple gold-standard assessments, alongside best estimate clinical judgment when possible in providing categorical groupings such as these. In addition, the absence of girls with ASD-O in this study limited conclusions regarding whether patterns found in girls with FXS-ASD extend to idiopathic ASD. Similarly, children with DS who met criteria for ASD based on the ADOS were excluded from the larger study. Specific cognitive and language requirements were also imposed as part of inclusion criteria into the study. This limits the generalizability of findings for this particular group (as well as for participants with FXS). Future studies should include a DS group with and without ASD to better understand the impact of ASD symptomatology on pragmatic language in this group, and whether similar differences exist to what is observed in FXS (i.e., it may be possible that while contingent discourse was a strength in this particular sample, individuals with DS with co-occurring ASD show difficulties in this aspect of language more similar to those with ASD-O and FXS-ASD). Future studies should also examine parent-child interaction styles with larger samples of fathers, to examine potential parent-specific effects that may differ between mothers and fathers (especially among mothers with the *FMR1* premutation). The reduced sample size of the longitudinal data available in this study also limited our ability to examine outcomes for some of the groups included in this study, though nonetheless provided valuable information on potential influences on pragmatic language growth in FXS, ASD, and DS over time, that should be replicated in larger samples. It is also important to note that the number of analyses increased the risk for Type 1 error overall. While reducing Type 2 error was the primary concern given the novelty of these unique data, the fact that questions were intended to guide next steps in future research, and the difficulties ascertaining a sample such as this, the risk for Type 1 error should be considered in interpreting findings.

Findings suggest several potential clinical implications. First, across idiopathic and syndromic ASD groups, we found that non-contingent language and perseveration represent a core and shared area of deficit. Clinical interventions designed to improve these deficits in ASD can facilitate social communicative skills and social competency more broadly. Many such interventions have been developed specifically for individuals with idiopathic ASD, and it will be important to examine the efficacy of these evidenced-based interventions among individuals with FXS-ASD. Further, the pragmatic impairments documented across these school-age groups could impact the ability to develop peer relationships, and as such, constitute important pragmatic skills that can be targeted in social communication interventions aimed at improving these specific language deficits throughout this age period. It will also be important to continue to provide parents with concrete strategies for how to best adapt their own pragmatic skills in the context of conversational interactions with their child, which may vary over the course of their child's development. This could be incorporated into already existing interventions in which parents serve as “social coaches” [e.g., (89)] for their children. Finally, that children in all groups showing increased pragmatic difficulties during unstructured interactions suggests that clinicians and researchers should incorporate both structured and unstructured assessment contexts in diagnostic evaluations and treatment plans.

## Data Availability Statement

The raw data supporting the conclusions of this article will be made available by the authors, without undue reservation.

## Ethics Statement

The studies involving human participants were reviewed and approved by University of North Carolina at Chapel Hill Institutional Review Board and Northwestern University Institutional Review Board. Written informed consent to participate in this study was provided by the participants' legal guardian/next of kin.

## Author Contributions

LB, GM, and ML contributed to conception and design of the study. LB and EL completed all data coding. LB performed the statistical analysis and wrote the first draft of the manuscript. All authors contributed to the article and approved the submitted version.

## Funding

This work was supported by grants from the National Institute of Child Health and Human Development (R01HD38819 and R01HD044935), the National Institute on Deafness and Other Communication Disorders (R01DC010191), and the National Institute of Mental Health (R01MH091131).

## Conflict of Interest

The authors declare that the research was conducted in the absence of any commercial or financial relationships that could be construed as a potential conflict of interest.

## Publisher's Note

All claims expressed in this article are solely those of the authors and do not necessarily represent those of their affiliated organizations, or those of the publisher, the editors and the reviewers. Any product that may be evaluated in this article, or claim that may be made by its manufacturer, is not guaranteed or endorsed by the publisher.
